# Identification of a Bidirectional Promoter from *Trichoderma reesei* and Its Application in Dual Gene Expression

**DOI:** 10.3390/jof8101059

**Published:** 2022-10-10

**Authors:** Xiaoxiao Wu, Fuzhe Li, Renfei Yang, Xiangfeng Meng, Weixin Zhang, Weifeng Liu

**Affiliations:** State Key Laboratory of Microbial Technology, Shandong University, No.72 Binhai Road, Qingdao 266237, China

**Keywords:** bidirectional promoter, filamentous fungus, cellulase, *sor* cluster

## Abstract

The cellulolytic filamentous fungus *Trichoderma reesei* has a strong capability in protein synthesis and secretion and is increasingly used as a fungal chassis for the production of heterologous proteins or secondary metabolites. However, bidirectional promoters that would significantly facilitate multiple genes’ expression have not been characterized in *T. reesei*. Herein, we show that a 767-bp intergenic region between two polyketide synthase encoding genes that were involved in the biosynthesis of the typical yellow pigment served as a bidirectional promoter in *T. reesei*. This region was shown to be able to drive the simultaneous expression of two fluorescence reporter genes when fused to each end. Quantitative RT-PCR analysis demonstrated that the driving strength of this bidirectional promoter from each direction reached about half of that of the commonly used promoter P*gpdA*. Moreover, the co-expression of two cellulase genes driven by this bidirectional promoter enabled *T. reesei* to produce cellulases on glucose and improved the total cellulase activities with cellulose Avicel as the carbon source. Our work identified the first bidirectional promoter in *T. reesei*, which would facilitate gene co-expression and find applications in synthetic biology using fungal systems.

## 1. Introduction

The filamentous fungus *Trichoderma reesei* has been used as an important industrial-scale cellulase producer for a long time due to its prominent capability in secreting a large quantity of cellulolytic enzymes as well as its safety and robustness in fermentation [1,2]. *T. reesei* is also increasingly applied as a microbial chassis for the production of heterologous enzymes and pharmaceutical proteins [2,3]. Moreover, it has been recently proved feasible to express heterologous biosynthetic gene clusters comprising multiple genes in a *T. reesei* strain with the inactivation of sorbicillinoid-type yellow pigment biosynthesis, which enables the fungus to convert waste biomass into secondary metabolites [3].

The production of either proteins or secondary metabolites requires promoters with appropriate strength and/or combinations for controlling gene expression in the host cells. Quite a few inducible or constitutive promoters have been identified and extensively used in *T. reesei* [4]. Most inducible promoters are from cellulase- or hemicellulase-encoding genes, most notably the promoter of *cbh1* that encodes the major extracellular cellulase CBHI [5]. Commonly-used constitutive promoters include *Aspergillus* P*gpdA*, *T. reesei* P*tef1*, P*cdn1*, P*pdc1*, and P*tcu1,* which respond to exogenously added copper [6]. Nonetheless, all of these promoters are monodirectional promoters (MDPs). While sufficient for single-gene expression, MDPs become limiting when applied for the co-expression of multiple genes since they are quite time-consuming, especially in eukaryotic microbes. In addition, consecutive genetic manipulation using MDPs for gene expression is prone to cause genetic instability, which is disadvantageous to efficient genetic construction and product biosynthesis.

In contrast, the application of bidirectional promoter (BDPs) helps to facilitate genetic engineering and expand expression flexibility. Natural BDPs have been found in almost all kingdoms, ranging from bacteria to mammals [7,8]. It has been shown that BDPs are always intergenic regions regulating the flanking two genes that encode proteins relevant to the same biological process. Up to now, only a few BDPs have been identified and characterized in filamentous fungi, most of which are present in *Aspergillus* and *Penicillium*. These BDPs were discovered in the intergenic regions between two divergently oriented genes involved in penicillin production [9,10], nitrogen metabolism [10,11,12], histone proteins [13], or hemicellulases synthesis [8], and some of them have been successfully used for dual gene co-expression [13]. However, no BDPs have been characterized in *T. reesei*, which limits the development of the multiple gene expression system in such an important eukaryotic chassis for the efficient synthesis of recombinant proteins or secondary metabolites.

The typical yellow pigment (sorbicillinoids) secreted by *T. reesei* is synthesized by the *sor* cluster, including two polyketide synthase encoding genes *sor1* and *sor2* [14]. These two genes are arranged as “head-to-head” and separated by a 767-bp intergenic region. In this study, we presented evidence that the intergenic region between *sor1* and *sor2* served as a BDP. We also quantified the promoter strength and used this BDP for the simultaneous co-expression of two cellulase genes in *T. reesei*.

## 2. Materials and Methods

### 2.1. Strains and Culture Conditions

*T. reesei* OE*ypr1*-Δ*sor1* [15] was used as the parent strain throughout this study. OE*ypr1*-Δ*sor1* was previously created via the overexpression of *ypr1* and the deletion of *sor1* in QM9414-∆*pyr4* [16], which is a derivative of *T. reesei* QM9414 (ATCC 26921). *T. reesei* cells were maintained on malt extract agar at 30℃. For the transcription and enzyme production analyses, *T. reesei* cells were cultured in Mandel–Andreotti (MA) medium [17] with 1% (*v/v*) glycerol as the carbon source for 36 h, collected, and washed thoroughly with MA medium without any carbon source. Equal mycelia were then transferred to the fresh medium with 1% (*w/v*) glucose or 1% (*w/v*) Avicel as the carbon source and cultivated for the indicated time period. *Escherichia coli* DH5α cells were used for routine plasmids construction. *E. coli* cells were cultured in lysogeny broth at 37 °C.

### 2.2. Construction of Plasmids and T. reesei Mutant Strains

To construct the expression cassette for *egfp* and *mCherry* under the control of P*sor*, their encoding regions were amplified from the plasmid P*tcu1*-*clp1*-*gfp*-T*trpC* [18] and P*tcu1*-*mCherry*-*h2b* [18], respectively. Three fragments, including P*sor* (767 bp) and two terminators T*trpC* (759 bp) and T*cbh2* (1 kb), were then amplified from the genomic DNA isolated from QM9414, respectively. The above five fragments were fused together via several rounds of overlap-extension PCR [19] to generate the expression cassette T*trpC*-*egfp*-P*sor*-*mCherry*-T*cbh2*. This cassette was subsequently inserted into the pUC19-*pyr4*-*sur* plasmid that contains two DNA fragments corresponding to approximately 2.3 kb and 2.3 kb of *pyr4* up- and downstream noncoding regions for *pyr4* locus integration and an expression cassette of *sur* that encodes an acetolactate synthase for resistance against sulfonylurea [20]. The resultant plasmid pUC19-*egfp*-P*sor*-*mCherry* was linearized and used to transform OE*ypr1*-Δ*sor1* to generate the recombinant strain *egfp*-P*sor*-*mCherry*. To construct the P*gpdA*-driven expression cassette for *egfp*, three DNA fragments, including P*gpdA*, *egfp*, and T*trpC,* were fused together and then ligated into pUC19-*pyr4*-*sur* to create pUC19-P*gpdA-egfp*. Similarly, to construct the expression cassette for *mCherry* under the control of P*gpdA*, three DNA fragments including P*gpdA*, *mCherry*, and T*cbh2* were fused together, followed by ligation into pUC19-*pyr4*-*sur* to create pUC19-P*gpdA-mCherry*. The plasmids pUC19-P*gpdA-egfp* and pUC19-P*gpdA-mCherry* were, respectively, linearized and transformed into OE*ypr1*-Δ*sor1* to generate the recombinant strain P*gpdA-egfp* and P*gpdA-mCherry*. To construct the expression cassette for two cellulase genes driven by P*sor*, the cDNA sequence of *ctcbh1* (GenBank Accession No. AM711862.1) was synthesized by the staff in GENEWIZ, Inc., and the full coding sequence of *eg1* together with its native terminator corresponding to downstream ~1 kb sequence (*eg1*-T*eg1*) were amplified from the genomic DNA of QM9414. Four fragments, including T*trpC*, *egfp*, P*sor,* and *eg1*-T*eg1,* were fused together and inserted into pUC19-*pyr4*-*sur* to create the expression plasmid pUC19-*ctcbh1*-P*sor*-*eg1*, which was subsequently linearized and transformed into OE*ypr1*-Δ*sor1* to yield the recombinant transformant *ctcbh1*-P*sor*-*eg1*. The transformation of *T. reesei* was performed as previously described [21]. Transformants were selected on a minimal medium for resistance to sulfonylurea (4 μg/mL). Anchored PCR was used to verify the correct integration events.

### 2.3. Fluorescence Microscopic Analysis

*T. reesei* spores were inoculated into minimal medium containing 1% (*w/v*) glucose and were cultured for 16 h at 30 °C in a shaking incubator. The fluorescence of mCherry and the GFP of the collected mycelia were detected using a research grade inverted NIKON TI-E fluorescence microscope (Nikon, Tokyo, Japan).

### 2.4. Enzyme Activity and Protein Analyses

Extracellular cellobiohydrolase activity was analyzed with 4-Nitrophenyl β-D-cellobioside (*p*NPC; Sigma-Aldrich, St. Louis, MO, USA) as a substrate by measuring the released *p*-nitrophenyl amount. The reaction was performed in 160 μL of reaction mixture with 80 μL of 50 mM sodium acetate buffer (pH4.8), 40 μL of substrate, and 40 μL of diluted culture supernatant. The mixture was then incubated at 50℃ for 30 min, and the reaction was stopped by the addition of 40 μL of 10% Na_2_CO_3_ (*w/v*). The amount of *p*-nitrophenyl is determined by measuring the absorbance at 420 nm. One unit (U) of *p*NPC activity is defined as the amount of enzyme releasing 1 μmol of *p*-nitrophenyl per minute. For endoglucanase activity, measurement was carried out in a 120 μL-reaction mixture containing 60 μL of culture supernatant and 60 μL of 0.5% carboxymethylcellulose sodium salt (CMC, Sigma Aldrich, St. Louis, MO, USA) dissolved in 50 mM sodium acetate buffer (pH 4.8) and was incubated at 50 °C for 30 min. With glucose as standard, the release of reducing sugar in the mixture was determined using the DNS method [22]. One unit of enzyme activity was defined as the amount of enzyme capable of releasing 1 μmol of glucose per minute. For protein identification, the target bands were excised from the SDS-PAGE gel and subjected to in-gel digestion with trypsin followed by liquid chromatography with tandem mass spectrometry (LC–MS–MS) on a MicrOTOF-Q II mass spectrometer (Bruker Daltonic, Billerica, MA, USA) connected to a prominence nano 2D (SHIMADZU, Kyoto, Japan) chromatography system. The MS raw data for each sample were combined and searched against the NCBI *T. reesei* protein sequence database using Mascot search engine version 2.3.01 software. LC–MS–MS and data analyses were performed by professional staff of Beijing Protein Innovation Co., Ltd. (Beijing, China).

### 2.5. RNA Extraction and Quantitative Real-Time PCR (qRT-PCR)

*T. reesei* mycelia were quickly frozen in liquid nitrogen and stored at −80 °C. Total RNA was extracted using TRIzol reagent (Sange Biotechnology, Shanghai, China), and gDNA was removed using TURBO DNA-free kit (Ambion, Austin, TX, USA) according to the instructions. The PrimeScript RT kit (Takara Bio, Kusatsu, Japan) was used for reverse transcription according to the instructions. Quantitative PCR was performed by the bio-RAD myIQ2 thermal cyclometer (BIO-RAD, Hercules, CA, USA). Data were analyzed using relative quantitative/comparative CT (ΔΔ CT) and normalized to the endogenous control (*actin*). Three biological replicates were performed for each analysis and for the results. Statistical analysis was performed by the student’s *t*-test.

### 2.6. Sequence Analysis

*T. reesei* nucleotide sequences were retrieved from the JGI database (https://mycocosm.jgi.doe.gov/Trire2/Trire2.home.html (accessed on 1 Sepetember 2019)). The presence of CpG island was predicted using online CpGFinder and CpGplot services available at websites of (http://www.softberry.com/ (accessed on 1 January 2021)) and (https://www.ebi.ac.uk/Tools/emboss/ (accessed on 1 January 2021)), respectively.

## 3. Results

### 3.1. The Intergenic Region between sor1 and sor2 Functions as a Bidirectional Promoter in T. reesei

**Figure 1:**
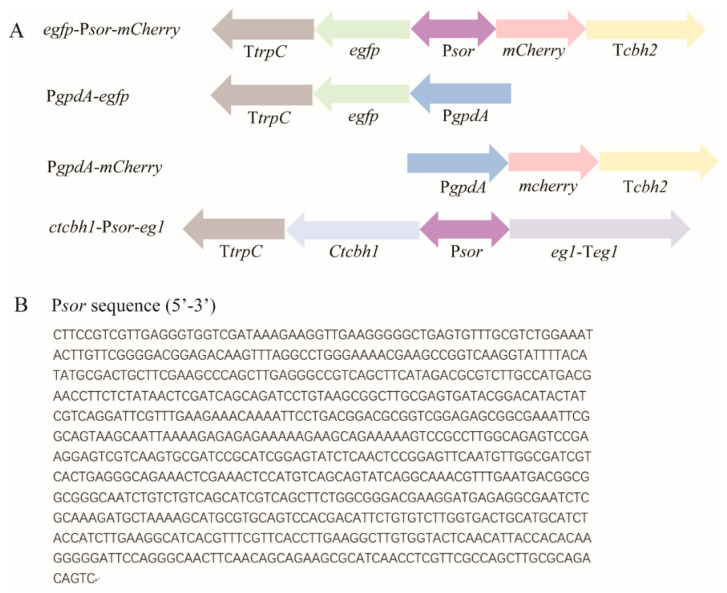
Schematic illustration of the expression cassettes incorporated into the indicated recombinant *T. reesei* strains, including *egfp*-P*sor*-*mCherry*, P*gpdA-egfp*, P*gpdA-mCherry,* and *ctcbh1*-P*sor*-*eg1* (**A**), and nucleotide sequence of P*sor* (**B**). The expression cassettes were, respectively, integrated into the *pyr4* locus of parental *T. reesei* strain. P*sor*: the 767 bp-intergenic region between *sor1* and *sor2*; P*gpdA*: the commonly used *gpdA* promoter from *Aspergillus*; T*trpC*: *trpC* terminator; and T*cbh2*: *cbh2* terminator. The sequence of P*sor* was retrieved from JGI database (https://mycocosm.jgi.doe.gov/Trire2/Trire2.home.html (accessed on 1 Sepetember 2019)).

**Figure 2:**
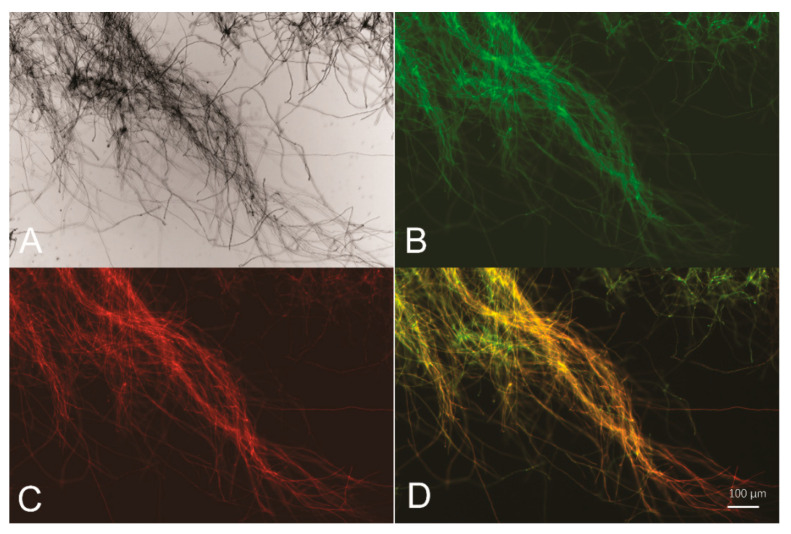
P*sor* is able to drive simultaneous expression of two reporter genes *egfp* and *mCherry***.** Bright-field (**A**), green fluorescence (**B**), red fluorescence (**C**), and merged fluorescence image (**D**) of *T. reesei* *egfp*-P*sor*-*mCherry* mycelia collected from glucose-containing cultures for 16 h.

To test whether the 767-bp intergenic region between *sor1* and *sor2* functions as a BDP, two reporter genes encoding a red fluorescence protein (mCherry) and a green fluorescence protein (GFP) were fused to either end of the intergenic sequence (hereafter named P*sor*), followed by transcription terminators T*cbh2* and Tt*rpc1*, respectively (Figure 1). The final expression cassette was integrated into the *pyr4* locus of the parental *T. reesei* strain via homologous recombination, and the resulting recombinant strain *egfp*-P*sor-mCherry* was cultured and subjected to fluorescence microscopic analyses. Based on the observations presented in Figure 2, P*sor* is able to simultaneously drive the expression of two reporter genes from both ends, indicating that P*sor* serves as a bidirectional promoter in *T. reesei*.

Sequence analyses showed that P*sor* shared some similarities with most reported BDPs in mammals [23], including a lack of the key core promoter element TATA box and the presence of a CpG island, which greatly contributed to the total high GC content (52%). In contrast to the obvious presence of a CpG island, poor information regarding the conserved binding motifs of transcriptional factors was predicted from P*sor* sequence.

### 3.2. Quantitative Determination of Promoter Strength of Psor

**Figure 3:**
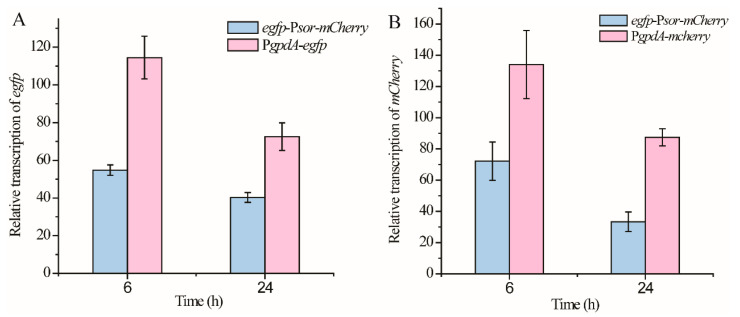
Comparison of the promoter strength from each direction of P*sor* with that of P*gpdA***.** Transcriptional analyses of *egfp* (**A**) from strains *egfp*-P*sor*-*mCherry* and P*gpdA-gfp* and transcriptional analyses of *mCherry* (**B**) from strains *egfp*-P*sor*-*mCherry* and P*gpdA-mCherry*. *T. reesei* cells were cultured in MA medium with 1% glucose as the carbon source. Values in this figure are the mean of three biological replicates. Error bars are the SD from these replicates.

Next, we compared the promoter strength from each direction of P*sor* with that of P*gpdA*, which is originally from *Aspergillus* and commonly used in *T. reesei* as a constitutive promoter. The respective expression cassette of *egfp* and *mCherry* driven by P*gpdA* was integrated into the *pyr4* locus of the parental *T. reesei* strain, to construct the reference strain P*gpdA*-*egfp* and P*gpdA-mCherry*, respectively (Figure 1). Whereas *egfp*-P*sor-mCherry* cells exhibited a similar signal strength of GFP or mCherry fluorescence with that observed from P*gpdA*-*egfp* or P*gpdA-mCherry*, their relative transcriptional levels of *egfp* or *mCherry* were further determined and compared using quantitative RT-PCR analyses. As shown in Figure 3A, the relative transcriptional level of *egfp* driven by P*sor* in the antisense direction reached up to ~50% of that driven by P*gpdA* during the early cultivation phase (6 h) and increased to ~62% when the cultivation time was extended to 24 h. Similar to *egfp*, the relative expression level of *mCherry* driven by P*sor* in the sense direction reached up to 53% and 38% of that driven by P*gpdA* after mycelial cultivation of 6 h or 24 h (Figure 3B). These results indicated that the driving strength of P*sor* from each end can come up to about half of that of P*gpdA.*

### 3.3. Co-Expression of Two Cellulase Genes Using Psor

**Figure 4:**
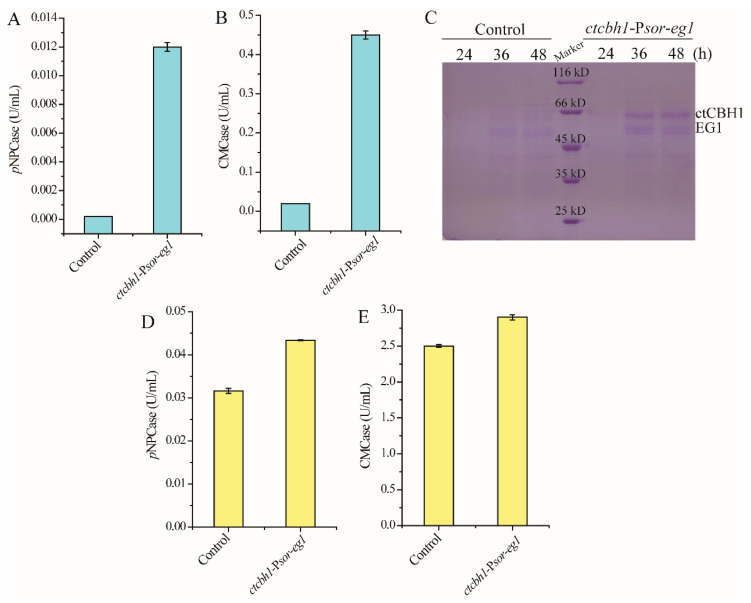
Co-expression of two cellulase genes using P*sor*. (**A**,**B**) Extracellular cellobiase (*p*NPCase, **A**) and endoglucanase (CMCase, **B**) activities of the strain *ctcbh1*-P*sor*-*eg1* cultured in MA medium with 1% glucose as the carbon source for 72 h. (**C**) SDS-PAGE analysis of extracellular supernatant of the strain *ctcbh1*-P*sor*-*eg1* cultured in MA medium with 1% glucose as the carbon source for 24–48 h. (**D**,**E**) Extracellular cellobiase (*p*NPCase, **D**) and endoglucanase (CMCase, **E**) activities of the strain *ctcbh1*-P*sor*-*eg1* cultured in MA medium with 1% Avicel as the carbon source for 120 h. The parental strain *T. reesei* OE*ypr1*-Δ*sor1* was used as the control strain. Values in this figure are the mean of three biological replicates. Error bars are the SD from these replicates.

P*sor* was further used for the co-expression of two cellulase genes: the major endoglucanase encoding gene *eg1* from *T. reesei,* and the gene encoding cellobiohydrolase I from another cellulolytic fungus *Chaetomium thermophilum* (*ctcbh1*) that has been shown to have higher catalytic activity than *T. reesei* cellobiohydrolase I [24]. In contrast with the parental strain, which did not produce any cellulase during cultivation on glucose, the strain *ctcbh1*-P*sor-eg1* displayed significant endoglucanase and cellobiohydrolase activities in the culture supernatant (Figure 4A,B), indicating that P*sor* simultaneously initiated the expression of *ctcbh1* and *eg1*. SDS-PAGE combined with mass spectrometric analysis verified the presence of *ct*CBHI and EGI in the culture supernatant (Figure 4C). Moreover, when *ctcbh1*-P*sor-eg1* was cultivated for 120 h with cellulose Avicel as the carbon source, the cellobiohydrolase and endoglucanase activities within the culture supernatant increased by 37% and 17%, respectively, as compared with the parental strain, demonstrating that the co-expression of two celluase genes driven by P*sor* contributes to improving total cellulase production (Figure 4D,E).

## 4. Discussion

Whereas BDPs are widespread in all kingdoms and have much application potential in the co-expression of genes, none have been identified in *T. reesei,* which serves as an important and robust fungal factory for the production of native or heterologous proteins or even heterologous secondary metabolites. Herein, we showed that P*sor,* within the *sor* cluster responsible for the synthesis of yellow pigment in *T. reesei*, functioned as a BDP and can be used for dual gene co-expression with two reporter genes and two cellulase genes as proofs of concept. Notably, the co-expression of two cellulase genes using P*sor* contributed to the improvement of total cellulase activities. We chose OE*ypr1*-Δ*sor1* as the parental strain, in which the yellow pigment production was completely abolished as a result of *sor1* deletion and therefore exhibited some preferred characters, including improved conidiation, the maintenance of cell wall integrity, and stress tolerance [15]. Particularly, the elimination of the yellow pigment from the culture supernatant greatly facilitates the downstream isolation of target products. Meanwhile, the overexpression of *ypr1* encoding the main activator for *sor* cluster [15] was expected to enhance the driving activity of P*sor*. OE*ypr1*-Δ*sor1* therefore served as an ideal parental strain for P*sor* application. Nonetheless, we also observed that even with the overexpression of *ypr1*, the promoter strength of P*sor* from each end reached only half of that as shown by the promoter P*gpdA*. Whereas this promoter strength may not satisfy the requirement from high-level gene expression, it is suitable for the synthesis of products whose excessive load would overburden the cellular machinery of the host, especially those with toxicity. It may also find applications in the synthesis of secondary metabolites that require the co-expression of multiple respective genes. The application of BDP would obviously facilitate genetic manipulation and therefore is helps to reduce costs.

Compared with several other reported BDPs from filamentous fungi with a length ranging from ~800 bp to 1200 bp [8,9,10,12,13], *T. reesei* P*sor* has a shorter length of 767 bp. Nonetheless, it is currently unclear whether the flanking regions of P*sor* (the coding sequences within *sor1* or *sor2*) are involved in the activation of *sor* genes, although the 767 bp-intergenic region is competent to drive the expression of two reporter genes and two cellulase genes. We have also noted that, whereas Ypr1 and Ypr2 have been demonstrated to act as the activator and repressor for *sor* genes, respectively [14], their binding sites as well as other *cis*-regulatory elements within P*sor* have not been identified yet. The future clarification of these key *cis*-regulatory elements and the regulatory mechanism to initiate the bidirectional transcription of P*sor* would contribute to reinforcing the promoter strength and reducing the promoter length.

## Data Availability

Not applicable.
